# Decrypting the Mitochondrial Gene Pool of Modern Panamanians

**DOI:** 10.1371/journal.pone.0038337

**Published:** 2012-06-04

**Authors:** Ugo A. Perego, Hovirag Lancioni, Maribel Tribaldos, Norman Angerhofer, Jayne E. Ekins, Anna Olivieri, Scott R. Woodward, Juan Miguel Pascale, Richard Cooke, Jorge Motta, Alessandro Achilli

**Affiliations:** 1 Sorenson Molecular Genealogy Foundation, Salt Lake City, Utah, United States of America; 2 Dipartimento di Biologia Cellulare e Ambientale, Università di Perugia, Perugia, Italy; 3 Instituto Conmemorativo Gorgas de Estudios de la Salud, Panama City, Panama; 4 Ancestry, Provo, Utah, United States of America; 5 Dipartimento di Biologia e Biotecnologie, Università di Pavia, Pavia, Italy; 6 Smithsonian Tropical Research Institute, Apdo, Ancón, Panama; University of Florence, Italy

## Abstract

The Isthmus of Panama–the narrow neck of land connecting the northern and southern American landmasses–was an obligatory corridor for the Paleo-Indians as they moved into South America. Archaeological evidence suggests an unbroken link between modern natives and their Paleo-Indian ancestors in some areas of Panama, even if the surviving indigenous groups account for only 12.3% of the total population. To evaluate if modern Panamanians have retained a larger fraction of the native pre-Columbian gene pool in their maternally-inherited mitochondrial genome, DNA samples and historical records were collected from more than 1500 volunteer participants living in the nine provinces and four indigenous territories of the Republic. Due to recent gene-flow, we detected ∼14% African mitochondrial lineages, confirming the demographic impact of the Atlantic slave trade and subsequent African immigration into Panama from Caribbean islands, and a small European (∼2%) component, indicating only a minor influence of colonialism on the maternal side. The majority (∼83%) of Panamanian mtDNAs clustered into native pan-American lineages, mostly represented by haplogroup A2 (51%). These findings reveal an overwhelming native maternal legacy in today's Panama, which is in contrast with the overall concept of personal identity shared by many Panamanians. Moreover, the A2 sub-clades A2ad and A2af (with the previously named 6 bp Huetar deletion), when analyzed at the maximum level of resolution (26 entire mitochondrial genomes), confirm the major role of the Pacific coastal path in the peopling of North, Central and South America, and testify to the antiquity of native mitochondrial genomes in Panama.

## Introduction

Most genetic studies that focus on the population dynamics of the first human groups that moved from North to South America across the Central American isthmus were based on data collected exclusively from surviving indigenous Native American groups. However, after the dramatic encounters with occupying Europeans starting about 500 years ago, the cultural, demographic, ethnic, and genetic landscapes of the Western Hemisphere were changed irreversibly. Today's Native American populations are a non-random remnant of the multitude of culturally and socially diverse groups, which developed over the ∼15 to ∼20 millennia that have elapsed since the first human groups moved from north-east Asia into America across Beringia [1]–[19]. Reconstructing the history of any people using modern-day populations is often challenging since current populations likely do not represent the full extent of variation that existed in earlier populations that may have changed vastly in composition in intervening years [20], [21]. This is also true for the uniquely-positioned, narrow geographic region of Panama, a pivotal cross-road corridor that connects America's northern and southern landmasses [22].

The extant Panamanian ethnic groups comprise 12.3% (417,559 of 3,405,813) of the Panamanian population [23], exhibit remarkable cultural resilience, and speak languages, which are historically related at different time depths [24]. The majority *ethnia* are the Ngäbe (also known as Ngöbe), Kuna (also called Guna) and Emberá; smaller *ethnia* are the Wounaán, Bribri, and Naso (also called Teribe). The speakers of Ngäbere are by far the most numerous group (>260,000 individuals). However, it is unknown whether pre-Columbian indigenous inhabitants represented the same cultural population as the modern ones. Archaeological records, as well as vegetation history derived from lake sediment studies in the country of Panama, show that, after the initial arrival towards the end of the last glaciation, some descendants of the earliest migrants remained on the isthmus, at least since Clovis times (13.2–12.8 Ky ago) [25], adapting their lifestyles to changing environmental and social conditions [24], [26]. The demographic scenario of Panama, as in every country in America, changed dramatically with the arrival of European settlers and their African slaves. Conquest impacted local populations differentially. Survival was best in mountainous and Caribbean regions where the Spanish colonizers had little interest in settling or did not have the resources to do so. In some areas (e.g. the central and western Caribbean and much of the Darién) they were rebuffed by the Native people, e.g. the Ngäbe and Kuna who descend from populations, which have lived continuously in some areas of Panama for a very long time.

At Spanish contact, the landscape was occupied by hundreds of sedentary farming communities arranged into small chiefdoms. In central Panama, where archaeological and paleoecological records are most complete, it is likely that the inhabitants of polities encountered by the Spanish were largely derived from much earlier populations residing in the same area [24]. As for the historical-linguistic data, a small vocabulary of ∼50 words of the Cueva language, which the Spanish say was spoken from central Panama to the Gulf of Urabá, is recorded in contact-period documents. It comprises some words that are cognate with modern Kuna and others with modern Waunaan [27]. This suggests that the Cuevan “language” may have been a *lingua franca* used for trade or that communities were multilingual [28], [29]. It also implies a degree of linguistic continuity in this area across Spanish contact. In western Panama, less than 10 native words were recorded by 16^th^ century chroniclers. Therefore it is impossible to determine whether these populations spoke recently extinct languages (Dorasque and Chánguena) or ancestral forms of the languages that are spoken today (Ngäbére, Buglére, and Naso). However, documentary evidence from after the 17^th^ century makes it very likely that forms of these languages were spoken in pre-Columbian times.

From a genetic point of view, the uniparental markers, Y chromosome and mitochondrial DNA (mtDNA), have been widely employed in the past leading to the identification of a high degree of differentiation in specific regions and/or ethnic groups, and highlighting the existence of genetic structures even in geographically proximate populations [30]–[33]. An example of this differentiation is the “Huetar deletion,” a peculiar control-region 6-bp deletion between nucleotide pairs (np) 106 and 111, which was identified by Santos and Barrantes [34] in a sample of Huetar (Costa Rica) whose now extinct language belongs to the Chibchan stock (*sensu* Constenla Umaña [29]). This corresponds to the *Msp*I site loss at np 104 within haplogroup A2 reported in several other Chibchan-speaking groups of Central America, including the Boruca, Bribri, Cabécar, Guaymí (Ngäbe and Bugle), Kuna, and Teribe [8], [35]–[38]. It was concluded that this mutation might have originated several millennia ago, when it was likely that lower Central American populations spoke ancient variants of Chibchan languages [29], and formed small and mobile social units. Only later did they congregate into tribes or chiefdoms, which, in spite of frequent inter- and intra-group conflict, continually traded with each other. In the most productive areas, especially on the Pacific side, a considerable degree of sedentism, and moderate to strong social ranking were achieved [24].

In brief, the all-important 16^th^ century is poorly understood. There is no evidence that the ancestral indigenous gene-pool of Panama (and lower Central America) was completely replaced. Some historical, linguistic and archaeological data point clearly towards continuity, at least in some areas of the Isthmus, deep in time and extending up to the Conquest era. If this is the case, the populations of modern Panama should have retained at least a fraction of the native pre-Columbian gene-pool, possibly to a variable extent, given the differential degree of geographical and genetic isolation of the different Panamanian communities during the past five centuries. In order to address this issue, we present a summary of the mtDNA variation from a large Panamanian sample obtained from the mixed general population living in Panama's urban areas as well as from autochthonous tribal groups. The analysis indicates that the study of mtDNA contributes to the understanding of evolutionary dynamics of the Native American population of this geographically-unique region.

## Results

### Comparing Molecular and Genealogical Data

**Figure 1:**
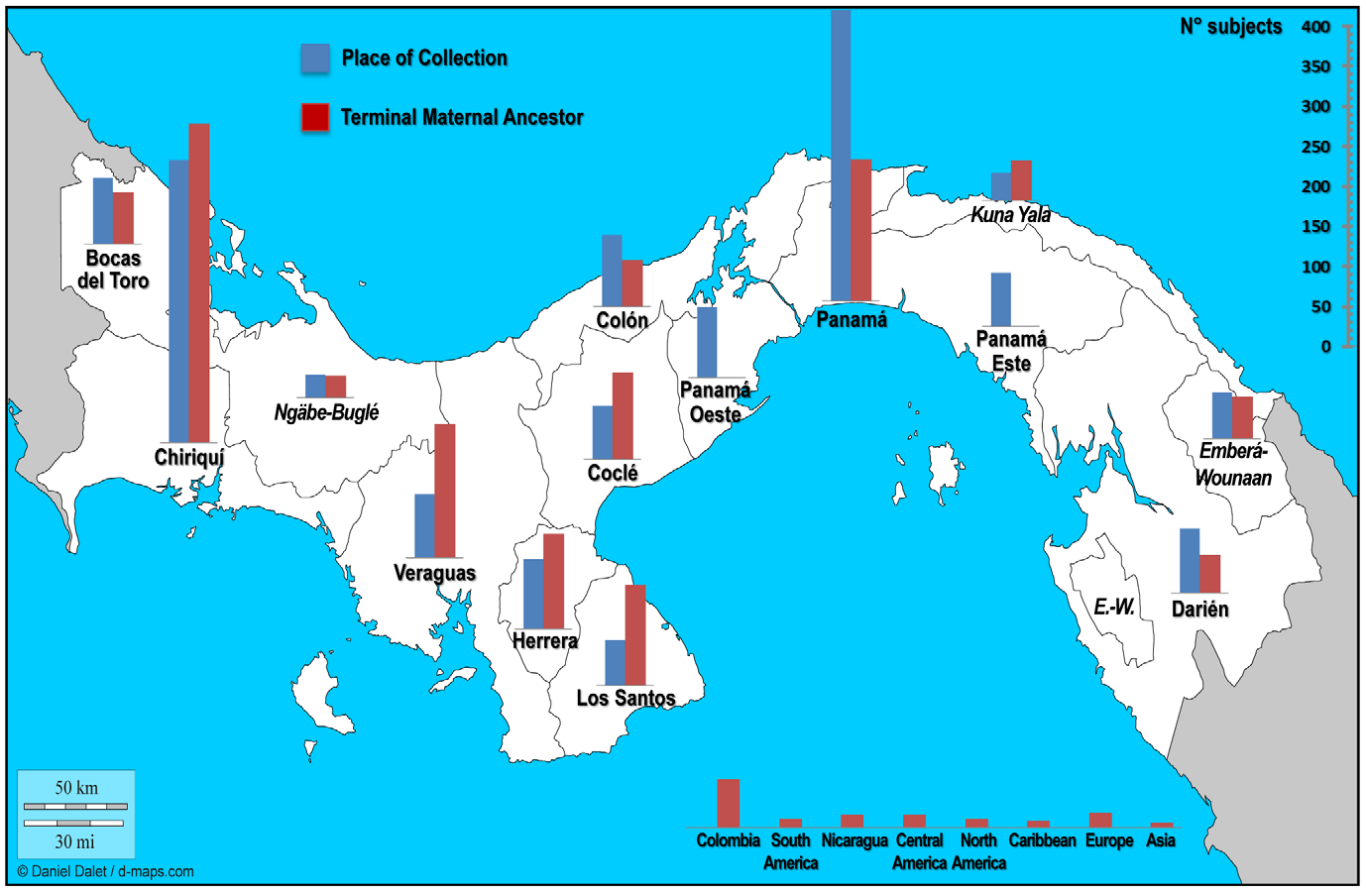
Distributions in Panama of the 1565 samples analyzed in this paper. Bars show both place of collection and terminal maternal ancestor (TMA) origin. This means the origin of the last known ancestor on the maternal side of the recorded pedigree.

MtDNA profiles for 1565 samples – collected in different Panamanian provinces or Native American comarcas (Figure 1) – were determined by sequencing 1150 base pairs (bps) from nucleotide position (np) 16000 to np 580 (Tables S1 and S2), thus covering the entire control region and including the three hypervariable segments (HVS-I: nps 16024–16383, HVS-II: nps 057-372, and HVS-III: nps 438-576). Excluding gaps and ambiguous sites, 227 polymorphisms and 865 invariable sites were identified in the control-region sequences with a nucleotide diversity (π) of 0.01144. The average number of nucleotide differences (k) between two randomly chosen sequences is 13.118. A total of 375 different haplotypes were observed, with an observed high diversity index (Hd = 0.971). These data confirm the efficacy of the sampling design, where related subjects were avoided. An accurate survey of mutational diagnostic motifs in the control region allowed the classification of mtDNAs into many haplogroups, sub-haplogroups, and paragroups, following the most updated mtDNA phylogeny and nomenclature [39] (Table S1).

Upon evaluating the genealogical data (with an average value of 3.13±0.87 ancestral generations) we found that 149 of the collected samples have a last known forebear on the maternal line from abroad, which means a non-Panamanian terminal maternal ancestor (TMA, Figure 1): 48.3% of them from South America (mostly from Colombia, 40.9%), followed by Central America (21.5%, half from Nicaragua), Europe (12.8%), North America (7.4%), Caribbean (6.0%), and Asia (4.0%). None of the TMAs were reported as being from Africa, probably due to the lack of genealogical records available during the slave trade era. It is worth noting that samples are all consistently assigned to haplogroups typical of the TMA area of origin (Table S1). Panamanian pedigrees were also analyzed in detail, and a search was conducted using the Sorenson Molecular Genealogy Foundation mtDNA database [40] in order to identify samples that might come from the same family (distant relatives) on the maternal side. A total of 123 cross-related samples (119 with a TMA from Panama, the other four from other countries) were identified: 42 pairs and 13 triplets (Table S1). Only one member for each of these extended families was retained. After excluding two samples with origins external to Panama (Costa Rica and Colombia), our final analyses were performed utilizing 1350 autochthonous and unrelated samples.

### Haplogroup Distribution

**Figure 2:**
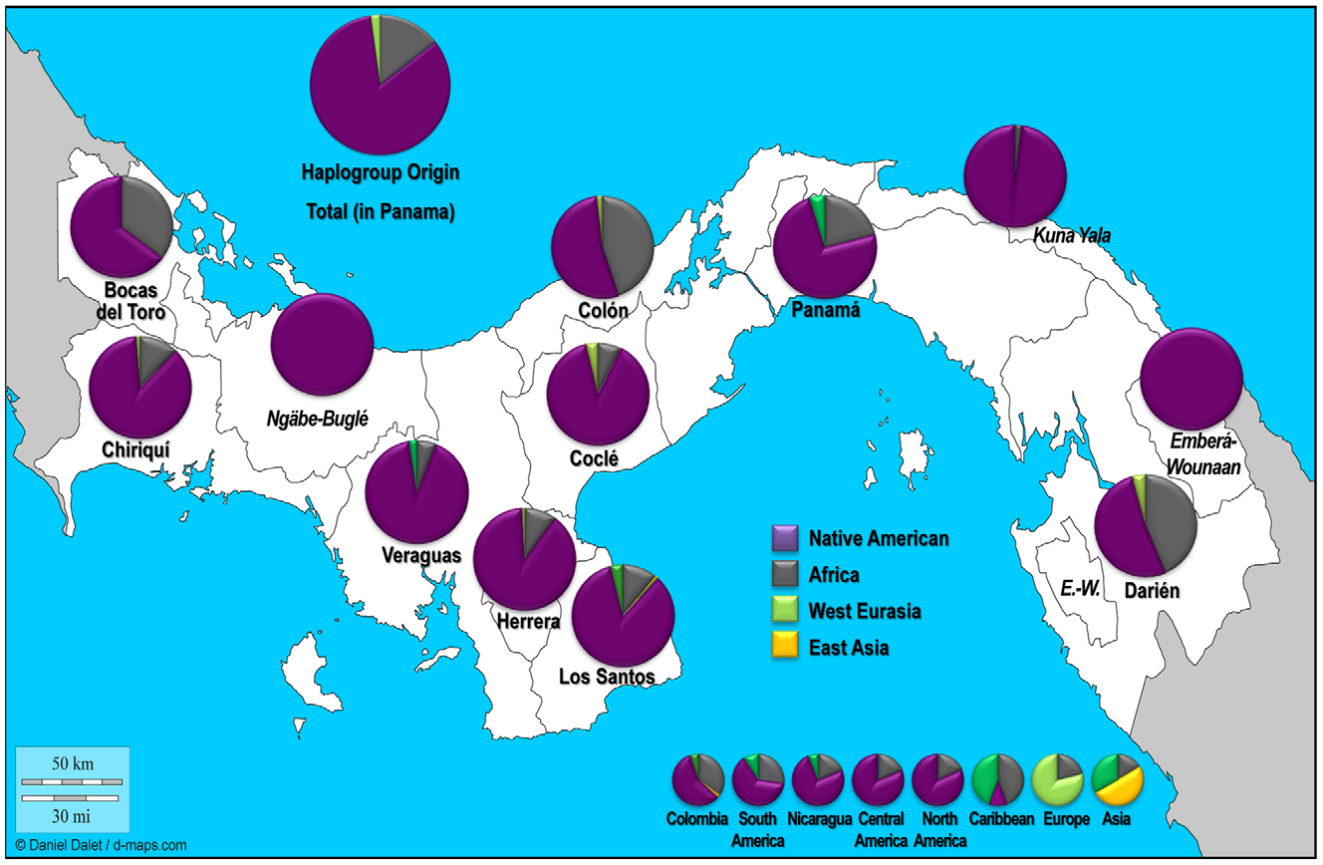
Spatial frequency (%) distributions of mtDNA haplogroups among the analyzed samples.

Approximately one fourth of the subjects with TMA from Panama were from the provinces of Chiriquí (28%), followed by Panamá (13%), and then Veraguas (12%) (Figure 1). These selected samples were assigned to more than a hundred different haplogroups and sub-haplogroups (Tables 1 and S1), grouped on the basis of their geographic/ethnic prevalence (Figure 2). A total of 83.5% of the analyzed lineages are of Native American origin [1], [6], [8], [41]. Not surprisingly, a significant percentage of sub-Saharan African [42]–[44] (14.4%) mtDNAs were also detected (although none of the genealogical records gathered indicated such ancestry), while a few Western Eurasian [45], [46] (2.1%) and East Asian [47], [48] clades (only one G1a1 in Los Santos) were identified. It is worth noting that the highest frequency of European haplogroups is in the Panamá province (5.1%), geographically coinciding with a spike in the African lineage signals (20.8%). However, sub-Saharan lineages are most common across the Caribbean (Bocas del Toro, 35.0%; Colòn, 45.6%) and in the easternmost province of Panama (Darién, 42.2%). All the four common “pan-American” haplogroups (A2, B2, C1, and D1) are well represented (83.5% overall), but none of the rare Native American haplogroups (D4h3a, X2a, and C4c) were observed. More than half of the Panamanians belong to haplogroup A2 (51.1%), the most common native lineage observed in Central America [49]. When comparing the haplogroup origin distribution between provinces and comarcas the difference is highly significant (χ^2^ *p-value*<0.0001), since, as expected, virtually all the samples with a TMA from the three comarcas (Ngäbe-Buglé, Emberá-Wounaan, and Kuna Yala) show Native American mtDNAs (99.2%). Intriguingly, the frequency of Native American haplogroups is quite different among the three comarcas (χ^2^ *p-value*<0.0001), with a prevalence of A2 in Kuna Yala (77.1%) and C1/D1 in Emberá-Wounaan (40.9%/6.8%), while the Ngäbe and Buglé together are about half A2 and half B2. Subsequently, when taking into account the distribution of indigenous populations in Panama, these data become even more interesting: e.g. high percentages of C1 and D1 (6.7% and 2.2%, respectively) stood out in the Darién province, where the Emberá and Wounaan indigenous people are most prevalent [23]. Actually, the distribution of Native American haplogroups is significantly different (χ^2^ *p-value* = 0.0004) between the eastern (southern) area of Panama (Panama Gulf provinces plus Colòn and Kuna Yala) and the western (northern) area. In fact, haplogroup C1 and D1 frequencies are noticeably higher in the eastern than the western area.

**Table 1:** Frequency distribution (%) of major haplogroups in Panama.

Province/Comarca	n°	A2	B2	C1	D1	Native	L0	L1	L2	L3	Africa	HV	JT	N1'2	U	West Eurasia	G	East Asia
Bocas del Toro	60	46.67	18.33	-	-	**65.00**	-	3.33	18.33	13.33	**35.00**	-	-	-	-	**0.00**	-	**0.00**
Chiriquí	378	60.05	24.07	1.06	1.32	**86.51**	1.59	4.76	4.23	1.59	**12.17**	0.79	0.53	-	-	**1.32**	-	**0.00**
Coclé	109	45.87	35.78	6.42	0.92	**88.99**	-	0.92	2.75	3.67	**7.34**	1.83	0.92	0.92	-	**3.67**	-	**0.00**
Colón	57	33.33	19.30	1.75	-	**54.39**	-	5.26	26.32	12.28	**43.86**	-	-	-	1.75	**1.75**	-	**0.00**
Darién	45	28.89	17.78	6.67	2.22	**55.56**	-	11.11	20.00	8.89	**40.00**	2.22	-	-	2.22	**4.44**	-	**0.00**
Herrera	116	61.21	24.14	3.45	-	**88.79**	-	-	4.31	6.03	**10.34**	-	-	-	0.86	**0.86**	-	**0.00**
Los Santos	122	56.56	24.59	2.46	0.82	**84.43**	0.82	-	2.46	7.38	**10.66**	3.28	-	-	0.82	**4.10**	0.82	**0.82**
Panamá	178	43.82	25.84	2.81	1.69	**74.16**	-	5.06	6.18	9.55	**20.79**	4.49	-	-	0.56	**5.06**	-	**0.00**
Veraguas	166	46.39	39.16	6.02	-	**91.57**	0.60	0.60	2.41	2.41	**6.02**	2.41	-	-	-	**2.41**	-	**0.00**
**Total Provinces**	**1231**	**51.34**	**26.73**	**3.01**	**0.89**	**81.97**	**0.65**	**3.17**	**6.26**	**5.36**	**15.43**	**1.79**	**0.24**	**0.08**	**0.41**	**2.52**	**0.08**	**0.08**
Emberá–Wounaan	44	15.91	36.36	40.91	6.82	**100.00**	-	-	-	-	**0.00**	-	-	-	-	**0.00**	-	**0.00**
Kuna Yala	48	77.08	12.50	8.33	-	**97.92**	-	-	2.08	-	**2.08**	-	-	-	-	**0.00**	-	**0.00**
Ngäbe-Buglé	27	48.15	48.15	3.70	-	**100.00**	-	-	-	-	**0.00**	-	-	-	-	**0.00**	-	**0.00**
**Total Comarcas**	**119**	**47.90**	**29.41**	**19.33**	**2.52**	**99.16**	**-**	**-**	**0.84**	**-**	**0.84**	**-**	**-**	**-**	**-**	**0.00**	**-**	**0.00**
**Grand Total**	**1350**	**51.04**	**26.96**	**4.44**	**1.04**	**83.48**	**0.59**	**2.89**	**5.78**	**4.89**	**0.84**	**1.63**	**0.22**	**0.07**	**0.37**	**2.30**	**0.07**	**0.07**

### Haplogroup A2

**Figure 3:**
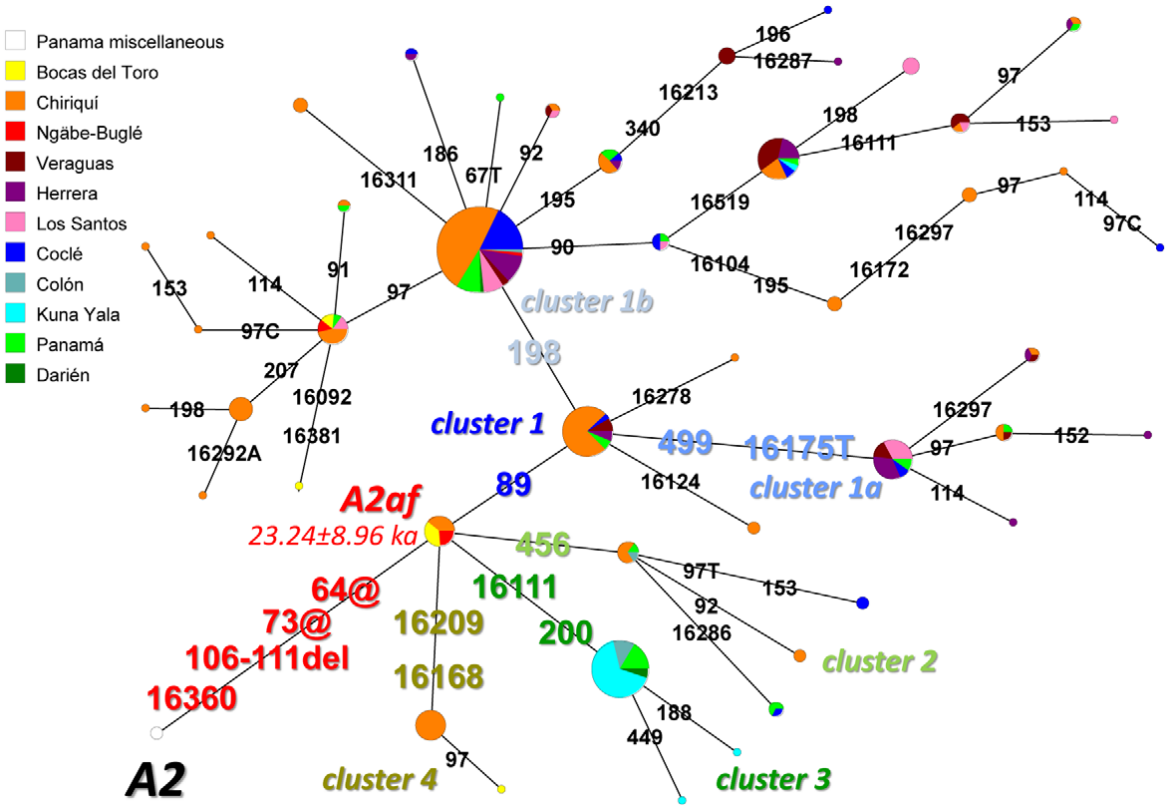
Network of A2af control-region haplotypes from Panama subdivided according to their geographic origin. The mutations on the connecting branches refer to the (revised) Cambridge reference sequence (rCRS) [52]. Markers of different clusters are in colors. Mutations are transitions unless the base change is explicitly indicated. Insertions, deletions and heteroplasmic mutations were excluded, with the notable exception of the 106–111 6 bp deletion. The size of each circle is proportional to the haplotype frequency and geographical origins are indicated by different colors. Coalescence ages of A2af and A2af1 are also reported using the control-region mutation rate reported by Soares *et al.* [50].

A total of 198 different A2 haplotypes (comprising 689 mtDNAs) were identified in Panama with the most common (53 mtDNAs) having the following mutational motif: 16111, 16223, 16290, 16319, 16360, 16362, 89, 106–111d, 146, 153, 198, 235, 263, 309.1C, 309.2C, 315.1C, and 522–523d. This and 79 other haplotypes were found to share the motif 64@, 73@, 106–111d, and 16360 (a total of 326 mtDNAs). The deletion of six base pairs (nps 106-111) was reported previously in the Huetars of Costa Rica and several other Chibchan-speaking groups of Central America. The additional control-region mutations detected in these haplotypes allow for a better classification of this particular A2 subclade, here preliminarily named A2af. A phylogenetic analysis of these mitochondrial genomes was performed through a network structure (Figure 3), resulting in four different sub-branches with a large prevalence of a particular one marked by the 89 transition. We employed the recent control-region mutation rate published by Soares et al. [50] to date the entire A2af clade at 23.24±8.96 ka.

### Entire Mitochondrial Genomes

**Figure 4:**
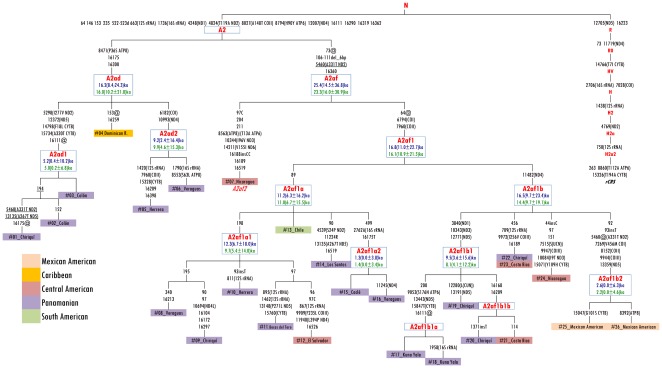
Phylogeny of complete mtDNA sequences belonging to haplogroups A2ad and A2af. The tree was rooted by using the reference sequence rCRS that is indicated for reading off sequence motifs. All sequences are new except for #04, #25 and #26 (Table 2). Mutations are shown on the branches; they are transitions unless a base is explicitly indicated. Suffixes indicate: reversions (@), transversions (to A, G, C, or T), indels (ins, del), gene locus, synonymous or non-synonymous changes. Recurrent mutations within the A2 branch are underlined. Any length variations in the C-stretch between nucleotides 303–315 and 16184–16193 were disregarded. Additional information regarding each mtDNA is available on Table 2. Coalescence times were calculated by converting into years [50] the averaged distance (*rho*, in blue) and the maximum likelihood (ML, in green) estimate, calculated by considering all the substitutions on the entire mitochondrial genome.

**Figure 5:**
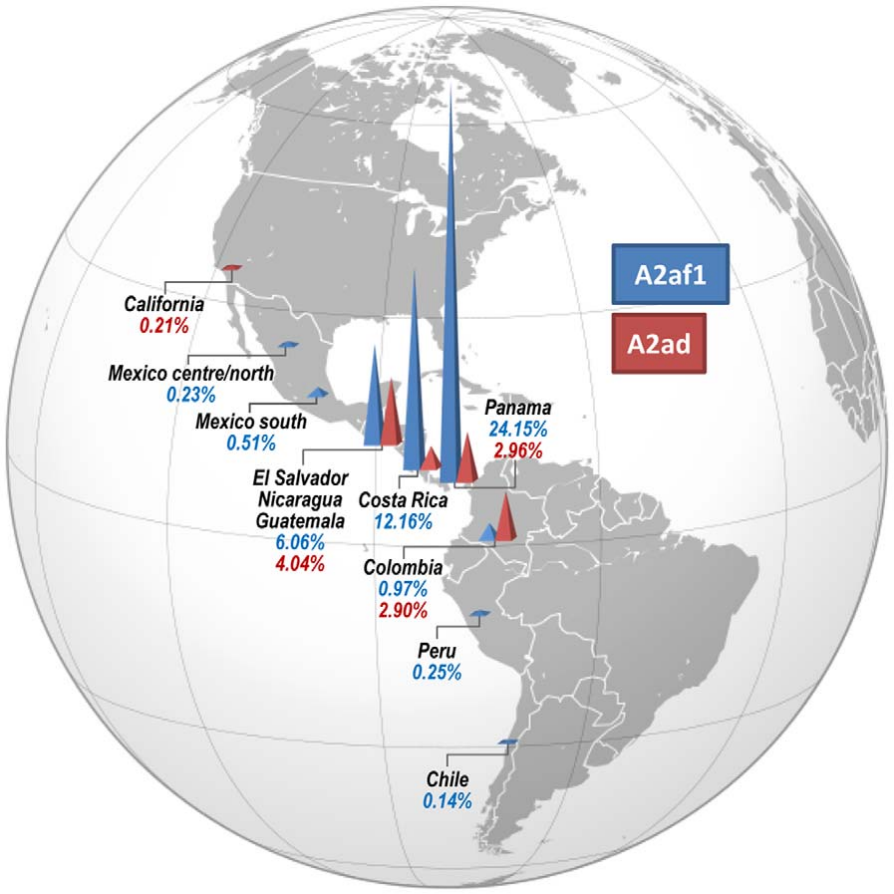
Spatial distribution of A2af and A2ad mtDNAs identified in general mixed populations. Exact values are listed in Table S3A.

To increase the resolution of our analyses, we also evaluated entire mitochondrial genomes. A total of 18 novel A2af mitochondrial genomes were completely sequenced (Table 2): 16 were collected in Panama (listed in Table S1); two others, collected in El Salvador and Chile, were already available in the SMGF dataset [40]. Overall, when looking at the TMA of these 18 samples, 12 were from Panama, two from Costa Rica, two from Nicaragua, one from El Salvador, and one from Chile. The evolutionary history of their mtDNAs was inferred by a parsimony approach and compared to two other Mexican American mtDNAs (MA145 and MA148) reported by Kumar et al. [51]. The latter two samples were misclassified as A2s, but they actually carry a clear A2af mutational motif. Through the phylogeny of Figure 4, rooted with the (revised) Cambridge Reference Sequence rCRS [52], we found that the Nicaraguan sequence #07 did not cluster either with A2af, or any of the known Old World A2 branches [39]. Its presence allows us to better define the A2af basal motif (73@, 106–111d, 5460, 16360), while the reversion at np 64, previously thought to be ancestral to the A2af (Figure 3), characterizes a major sub-branch A2af1 together with two coding-region transitions at nps 6794 and 7960. On the other hand, the Nicaraguan sequence #07 most likely indicates an additional and very rare Native American A2af sub-clade, here named A2af2. Concerning the major cluster of the phylogeny, we were able to date the terminal maternal ancestor of haplogroup A2af1 at ∼17 ka ago. The complete sequence analysis confirms the main sub-branching (A2af1a) marked by the control-region mutation 89, already detected in the control-region network analysis, but reveals also an additional sister branch defined only by a coding region transition at np 11482, here named A2af1b. Surprisingly, a member of A2af1a is from Chile and was one of the two A2af mtDNAs from South America that were found in the SMGF control-region database, the other one was from Colombia. Considering the overlapping patterns of Native lineages (obtained in some recent papers [5], [49]) when comparing haplogroup frequency distributions from general-mixed populations to that of Native American tribes or communities, we proceeded to analyze the incidence of A2af among the 79,928 records (as of March 2^nd^, 2012) of the SMGF database (Table S3A). Figure 5 shows that this peculiar haplogroup is detected at low frequencies along the Pacific coast and the western side of the Andes, but with great incidences in lower Central America, having its highest peaks in Costa Rica (12.16%) and Panama (24.15%). Previously, A2's HVS-I haplotypes carrying the 6-bp deletion were observed almost exclusively in Central America (Table S3B), especially (frequency >10%) among the Huetars (56%) [36] and the Bribri (74%) [38] of Costa Rica, and in Panama among the Ngäbe (9–12%) [32] [41] and the Kuna (53%) [41].

**Table 2:** Sources and haplogroup affiliation for the A2ad and A2af complete mtDNA sequences.

ID (Fig. 4)	Place of Collection	TMA Origin	Clade	GenBank	Original ID
**#01**	Chiriquí	Panama (Chiriquí)	A2ad1	JQ965966	S-947838
**#02**	Colón	Panama (Colón)	A2ad1	JQ965967	S-947655
**#03**	Colón	Panama (Colón)	A2ad1	JQ965968	S-947625
**#04**	Dominican R	Dominican R	A2ad	EF079873	#7_A2(Tor22)
**#05**	Herrera	Panama (Herrera)	A2ad2	JQ965969	S-996787
**#06**	Panamá	Panama (Veraguas)	A2ad2	JQ965970	S-915431
**#07**	Panamá	Nicaragua	A2af	JQ965971	S-915367
**#08**	Panamá	Panama (Veraguas)	A2af1a1	JQ965972	S-992683
**#09**	Chiriquí	Panama (Chiriquí)	A2af1a1	JQ965973	S-948115
**#10**	Darién	Panama (Herrera)	A2af1a1	JQ965974	S-943682
**#11**	Bocas del Toro	Panama (Bocas del Toro)	A2af1a1	JQ965975	S-948541
**#12**	El Salvador	El Salvador	A2af1a1	JQ965976	S-635621
**#13**	Chile	Chile	A2af1a	JQ965977	S-686714
**#14**	Los Santos	Panama (Los Santos)	A2af1a	JQ965978	S-996672
**#15**	Chiriquí	Panama (Coclé)	A2af1a2	JQ965979	S-947916
**#16**	Veraguas	Panama (Veraguas)	A2af1a2	JQ965980	S-992645
**#17**	Panamá	Panama (Kuna Yala)	A2af1b1a	JQ965981	S-937137
**#18**	Panamá	Panama (Kuna Yala)	A2af1b1a	JQ965982	S-915331
**#19**	Bocas del Toro	Panama (Chiriquí)	A2af1b1	JQ965983	S-948757
**#20**	Chiriquí	Panama (Chiriquí)	A2af1b1b	JQ965984	S-947228
**#21**	Bocas del Toro	Costa Rica	A2af1b1b	JQ965985	S-948517
**#22**	Chiriquí	Panama (Chiriquí)	A2af1b	JQ965986	S-948533
**#23**	Chiriquí	Costa Rica	A2af1b	JQ965987	S-948446
**#24**	Chiriquí	Nicaragua	A2af1b	JQ965988	S-948713
**#25**	US (Texas)	Mexican American	A2af1b2	HQ012106	MA148
**#26**	US (Texas)	Mexican American	A2af1b2	HQ012104	MA145

The second most common A2 subgroup in Panama is defined by the control-region mutational motif 16175–16300, representing about 3% of modern Panamanians. This new clade, named A2ad, was found in the SMGF database (and in the literature, Table S3B) in mostly the same countries as A2af, but at lower frequencies (Figure 5). This similarity is both spatial and temporal. In fact, A2ad was dated at approximately 16 ka ago, by looking at the entire sequence variation of five randomly selected Panamanian mtDNAs and one published Dominican sample [1] belonging to this clade (Figure 4).

## Discussion

According to the last official census [23], less than 420,000 Panamanians (12.3%) recognize themselves as Native American, while about 313,000 declare to be Afro-descendants (9.2%). Most of the Native American population live in the three comarcas (95%) where people of African descent are less than 1%; thus when looking at the provinces' general population, indigenous percentage decreases to 7.1%, while Afro-descendants remain almost the same (9.7%). In this study, we provided a comprehensive overview of the mitochondrial gene pool of Panamanians, with ∼80% of mtDNAs belonging to the native lineages A2, B2, C1, and D1, with A2 being the predominant clade. A similar extreme pattern was actually observed in another Central American country, El Salvador [53], where the African lineages are virtually absent (only one mtDNA of sub-Saharan origin). In Panama, the sub-Saharan component consists of 57 haplogroups and sub-haplogroups accounting for 14.4% of modern Panamanians, mostly identified in the Pacific provinces of Panamà and Darién and the Caribbean provinces of Colón and Bocas del Toro, where the Atlantic slave trade and more recent migrations from the Caribbean islands and northern Colombia clearly had a more relevant demographic impact [54], [55]. The oldest African population stems from African slaves since all Spanish settlements would have had some. There would have been concentrations around early towns such as Panama la Vieja, Natá, Los Santos, Nombre de Dios and Portobelo and also at mines like the Concepción mine in Caribbean Veraguas where several thousand slaves were employed in the late 16^th^ century [56]. African lineages found in Darién (Figure 2) are likely to derive directly from slaves who escaped Spanish rule and were known as “*cimarrones*” although the more recent immigration of people of African descent from northernmost Colombia should be taken into account. This population and others settled between Colón and Kuna Yala, and along the western Caribbean, speak Spanish, and are culturally different from the descendants of more recent immigrants from French- and English-speaking islands in the Caribbean who came to work on the Panama Canal. Another group of African people are the “turtlers” (*tortugueros*) who settled in the western Caribbean mostly from English-speaking islands such as Providence and the Cayman Islands. The cemetery at Drago on Isla Colón has grave stones of people born in Cayman in 1790 AD [57].

In conclusion, our molecular data reveal an overwhelming native maternal legacy in the modern Panamanian population. It seems that the Spanish conquistadores and additional more recent European demographic influences did not contribute significantly to today's genetic composition of Panama, at least with regard to the maternal side. These data are in contrast with the overall concept of personal origin shared by many Panamanians. Moreover, through the micro-phylogeographic approach proposed with the current Panamanian dataset, we were able to confirm a distinct sub-structure of native lineage distributions. Haplogroups C1 and D1 harbour much higher frequencies in the eastern (southern) area of Panama, particularly in Darién, where the Emberá and Wounaan indigenous people now prevail. A2 is prevalent in Kuna Yala, where 62.5% of people carry the previously called Huetar deletion (see introduction), thus belonging to the newly defined A2af haplogroup. Complete sequence analyses of this and the other most common A2 sub-lineage in Panama (A2ad) places their founder ages at more than 10 ka ago, highlighting an ancient expansion and settlement through this area. These two lineages are not found in North America and along the Atlantic coast, but can be observed at low frequencies as far south as Peru and Chile (Valparaiso). Considering the most recently accepted age estimate for haplogroup A2 in the American continent as a whole at 15–19 ka ago [5], [7], [51] and as a proxy for the time of expansion of Paleo-Indians into the Americas, it can be suggested that the initial settlement of Panama occurred fairly rapidly after the initial colonization of the American continent. These data fully support the hypothesis that the Pacific coast was the major entry point and diffusion route for the earliest human settlers. Moreover, the antiquity and high frequency of subclade A2af provides evidence of the existing mitochondrial DNA legacy between modern Panamanians and America's first inhabitants.

## Materials and Methods

### Ethics Statement

All experimental procedures and individual written informed consent, obtained from all donors, were reviewed and approved by the Comité Nacional de Bioética de la Investigación of Panama and by the Western Institutional Review Board, Olympia, Washington (USA).

### Specimen Collection

A total of 1565 saliva samples were collected from healthy unrelated individuals in different areas of Panama in collaboration with the Gorgas Memorial Institute for Health Studies. The field sampling was undertaken with the kind help of local assistants.

Mouthwash rinsing was the primary method of biological specimen collection using a method (GenetiRinse™) that comprises the use of 10cc of commercially available mint-flavored Scope™ mouthwash in a 15cc volume leak-free Nalgene™ plastic container. Participants swished the 10cc of mouthwash for 45 seconds and then spat the mouthwash back into its original container. Participants were asked to abstain from eating or drinking for at least 30 minutes prior to the mouthwash rinse. Total DNA was extracted from mouthwash using standard commercial kits (Qiamp DNA Blood Maxi Kit, Qiagen) and stored at −20°C.

### Analysis of mtDNA Control-Regions

The first step of the mtDNA molecular analyses consisted of DNA PCR amplification of the control region using the primers listed in Table S2. After PCR, fragments were purified using the ExoSAP-IT® enzymatic system (Exonuclease I and Shrimp Alcaline Phosphatase, GE Healthcare) and Cycle Sequencing was performed by application of ABI Prism™ BigDye Terminator chemistry. A protocol including three forward and three reverse sequencing primers was used (Table S2). Primer redundancy is employed particularly for mtDNAs harbouring the transition T16189C, which creates a poly-C tail that causes premature termination of the sequencing reaction. The additional reverse primers solve the problem by completing the sequence information with multiple reads.

Electropherograms were aligned, assembled, and compared using the software Sequencher™ 5.1 (Gene Codes). Finally the mutational differences relative to the Cambridge reference sequence (rCRS) [52] were accurately analyzed in order to identify mutational motifs for haplogroup classification following the most updated human mitochondrial phylogeny [39].

The median-joining network of control-region haplotypes observed in 326 A2af mtDNAs was constructed by using the Network 4.6 software program (http://www.fluxus-engineering.com). The time estimates option was employed to measure the age of an ancestral node in mutational units. This mutational age was then converted into years by the recent control-region mutation rate published by Soares et al. [50].

### Analysis of Entire Mitochondrial Genomes

Sequencing of entire mtDNA genomes belonging to haplogroups A2ad and A2af were performed as previously described [5], [58]. In order to obtain coalescence times, we directly calculated the average distance (ρ) of the haplotypes of a clade to the respective root haplotype, accompanied by a heuristic estimate of the standard error (σ) calculated from an estimate of the genealogy. PAML 4.5 [59] was used to calculate maximum likelihood (ML) estimates, assuming the HKY85 mutation model (with indels ignored, as usual) with gamma-distributed rates (approximated by a discrete distribution with 32 categories) and three partitions: HVS-I (positions 16051–16400), HVS-II (positions 68–263), and the remainder. These calculations were performed on entire mtDNA haplotypes (excluding the mutations 16182C, 16183C, 16194C and 16519). Mutational distances were converted into years using the substitution rate for the entire molecule of about one mutation every 3,624 years [50].

## Supporting Information

Table S1 Control-region haplotypes (relative to rCRS) and haplogroup/sub-haplogroup classification (based on PhyloTree, Built 14) of the 1565 mtDNAs collected in Panama and deposited in the SMGF database (http://www.smgf.org).
(XLS)

Table S2 Oligonucleotides used for amplifying and sequencing the entire control region.
(PDF)

Table S3 Distribution of haplogroups A2ad and A2af (**a**) in the SMGF database (general mixed populations) and (**b**) in the literature (native samples and forensic/population cohorts).
(PDF)
