# Supplementary material for: Decrypting the Mitochondrial Gene Pool of Modern Panamanians
Source: PLoS One. 2012 Jun 4;7(6):e38337. doi: 10.1371/journal.pone.0038337 (PMC3366925; doi:10.1371/journal.pone.0038337)
Supplement: Table S2 — Oligonucleotides used for amplifying and sequencing the entire control region. (PDF) [file pone.0038337.s002.pdf]

Table S2. Oligonucleotides used for amplifying and sequencing the entire control region.

**Name [a]**	**Length (nt)**	**Sequence (5'→3')**	**Tm (°C)**
**Amplification**			
15748 for	21	tctaacctgaatcggaggaca	59.68
765 rev	21	gtgcttgatgcttgttccttt	59.37
**Sequencing**			
15819 for	22	acttcacaacaatcctaatcct	54.54
16213 for	20	gcaatcaaccctcaactatc	54.69
48 for	20	catttggtattttcgtctgg	55.68
58 rev	20	aataccaaatgcatggagag	55.17
497 rev	22	ggggttgtattgatgagattag	55.30
653 rev	20	cctatttgtttatggggtga	55.04

[a] It corresponds to the first nucleotide position (at 5'), numbered according to rCRS.
