# Supplementary material for: Decrypting the Mitochondrial Gene Pool of Modern Panamanians
Source: PLoS One. 2012 Jun 4;7(6):e38337. doi: 10.1371/journal.pone.0038337 (PMC3366925; doi:10.1371/journal.pone.0038337)
Supplement: Table S3 — Distribution of haplogroups A2ad and A2af (a) in the SMGF database (general mixed populations) and (b) in the literature (native samples and forensic/population cohorts). (PDF) [file pone.0038337.s003.pdf]

Table S3A. Distribution of haplogroups A2ad and A2af in the SMGF database (general mixed populations). Haplogroup classification is based on entire control-region haplotypes.

Country	A2af	A2ad	Sample size
California (US)		1	479
Mexico Centre/north	3		1278
Mexico South	5		977
Guatemala	2	1	24
El Salvador	1		25
Costa Rica	9	1	74
Nicaragua	1	1	21
Panama	326	40	1350
Colombia	3	6	207
Chile	2		730
Peru	5		2005

Table S3B. Distribution of haplogroups A2ad and A2af in the literature (native samples and forensic/population cohorts). Haplogroup classification is based mostly on HVS-I haplotypes.

Country	A2af	A2ad	Sample size	Ethnic group	Reference
Mexico	3	-	270		[1]
Mexico	1	-	59	Nahuas	[2]
Cuba	-	5	245		[3]
Dominican Rep.	1	-	83		[4]
Guatelama	1? (aDNA)	-	12		[5]
El Salvador	2	2	90		[6]
Costa Rica	23	-	31	Bribri	[7]
Costa Rica	15	-	27	Huetar	[8]
Costa Rica	4	1	90		[9]
Costa Rica	1	-	44		[10]
Panama	1	-	63	Kuna	[11]
Panama	4	-	46	Ngäbe	[12]
Panama	18	-	34	Kuna	[13]
Panama	9	-	77	Ngäbe	[13]
Panama	1	-	57	Wounaan	[13]
Venezuela (Pueblo Llano)	5	-	219		[14]
Colombia	1	-	20	Páez	[15]
Brazil	1	-	172		[16]
Argentina	1	-	384		[17]
Uruguay	1	-	120		[18]

Supporting References

1. Guardado-Estrada M, Juarez-Torres E, Medina-Martinez I, Wegier A, Macías A, et al. (2009) A great diversity of Amerindian mitochondrial DNA ancestry is present in the Mexican mestizo population. J Hum Genet 54: 695-705.
2. Sandoval K, Buentello-Malo L, Peñaloza-Espinosa R, Avelino H, Salas A, et al. (2009) Linguistic and maternal genetic diversity are not correlated in Native Mexicans. Hum Genet 126: 521-531.
3. Mendizabal I, Sandoval K, Berniell-Lee G, Calafell F, Salas A, et al. (2008) Genetic origin, admixture, and asymmetry in maternal and paternal human lineages in Cuba. BMC Evol Biol 8: 213.
4. Tajima A, Hamaguchi K, Terao H, Oribe A, Perrotta VM, et al. (2004) Genetic background of people in the Dominican Republic with or without obese type 2 diabetes revealed by mitochondrial DNA polymorphism. J Hum Genet 49: 495-499.
5. Boles TC, Snow CC, Stover E (1995) Forensic DNA testing on skeletal remains from mass graves: a pilot project in Guatemala. J Forensic Sci 40: 349-355.
6. Salas A, Lovo-Gómez J, Alvarez-Iglesias V, Cerezo M, Lareu MV, et al. (2009) Mitochondrial echoes of first settlement and genetic continuity in El Salvador. PLoS One 4: e6882.
7. Santos M, Barrantes R (1994) Direct screening of a mitochondrial DNA deletion valuable for Amerindian evolutionary research. Hum Genet 93: 435-436.
8. Santos M, Ward RH, Barrantes R (1994) mtDNA variation in the Chibcha Amerindian Huetar from Costa Rica. Hum Biol 66: 963-977.
9. Morera B (2002) Análisis del polimorfismo del ADNmt en la población general de Costa Rica: un asunto pendiente. Revista Latinoamericana de Derecho Médico y Medicina Legal 7: 21-30.
10. Castrì L, Otárola F, Blell M, Ruiz E, Barrantes R, et al. (2007) Indentured migration and differential gender gene flow: the origin and evolution of the East-Indian community of Limón, Costa Rica. Am J Phys Anthropol 134: 175-189.
11. Batista O, Kolman CJ, Bermingham E (1995) Mitochondrial DNA diversity in the Kuna Amerinds of Panamá. Hum Mol Genet 4: 921-929.
12. Kolman CJ, Bermingham E, Cooke R, Ward RH, Arias TD, et al. (1995) Reduced mtDNA diversity in the Ngöbé Amerinds of Panamá. Genetics 140: 275-283.
13. Tamm E, Kivisild T, Reidla M, Metspalu M, Smith DG, et al. (2007) Beringian standstill and spread of Native American founders. PLoS ONE 2: e829.
14. Gómez-Carballa A, Ignacio-Veiga A, Alvarez-Iglesias V, Pastoriza-Mourelle A, Ruíz Y, et al. (2012) A melting pot of multicontinental mtDNA lineages in admixed Venezuelans. Am J Phys Anthropol 147: 78-87.
15. Torres MM, Bravi CM, Bortolini MC, Duque C, Callegari-Jacques S, et al. (2006) A revertant of the major founder Native American haplogroup C common in populations from northern South America. Am J Hum Biol 18: 59-65.
16. Guerreiro-Junior V, Bisso-Machado R, Marrero A, Hünemeier T, Salzano FM, et al. (2009) Genetic signatures of parental contribution in black and white populations in Brazil. Genet Mol Biol 32: 1-11.
17. Catelli ML, Alvarez-Iglesias V, Gómez-Carballa A, Mosquera-Miguel A, Romanini C, et al. (2011) The impact of modern migrations on present-day multi-ethnic Argentina as recorded on the mitochondrial DNA genome. BMC Genet 12: 77.
18. Pagano S, Sans M, Pimenoff V, Cantera AM, Alvarez JC, et al. (2005) Assessment of HV1 and HV2 mtDNA variation for forensic purposes in an Uruguayan population sample. J Forensic Sci 50: 1239-1242.
